# Attachment-oriented psychological intervention for couples facing breast cancer: protocol of a randomised controlled trial

**DOI:** 10.1186/2050-7283-2-19

**Published:** 2014-07-14

**Authors:** Anne Nicolaisen, Dorte G Hansen, Mariët Hagedoorn, Henrik E Flyger, Nina Rottmann, Per Nielsen, Katrine Søe, Anne E Pedersen, Christoffer Johansen

**Affiliations:** National Research Centre for Cancer Rehabilitation, Research Unit of General Practice, University of Southern Denmark, J. B. Winsløws Vej 9A, Odense, C DK-5000 Denmark; Department of Health Sciences, Health Psychology Research Section, University Medical Center Groningen, University of Groningen, Ant Deusinglaan 1, Groningen, 9713 AV The Netherlands; Department of Breast Surgery, Herlev University Hospital, Herlev Ringvej 75, Herlev, DK-2730 Denmark; Authorised privately practicing psychologist, Odense, DK-5000 Denmark; Department of Breast Surgery, Odense University Hospital, Sdr. Boulevard 29, Odense, C DK-5000 Denmark; Department of Breast Surgery, Ringsted Hospital, Bøllingsvej 30, Ringsted, DK-4100 Denmark; Danish Cancer Society Research Center, Survivorship, Danish Cancer Society, Strandboulevarden 49, Copenhagen, DK-2100 Denmark

**Keywords:** Breast cancer, Partners, Psychological intervention, Attachment, RCT

## Abstract

**Background:**

There is evidence that both breast cancer patients and their partners are affected emotionally, when facing a breast cancer diagnosis. Several couple interventions have been evaluated, but there is a need for couple intervention studies with a clear theoretical basis and a strong design. The Hand in Hand intervention is designed to enhance interdependent coping in the couples and to address patients and partners that are both initially distressed and non-distressed.

**Methods:**

The Hand in Hand study is a randomised controlled trial among 199 breast cancer patients and their partners. Couples were randomised to 4-8 couple sessions with a psychologist in addition to usual care, or to usual care only, approximately 2 months after the patients’ primary surgery date. The intervention was delivered within 3 months, and outcomes were assessed prior to randomisation and 5 and 10 months after primary surgery date. The primary outcome is patients’ cancer-specific distress at the 5-month follow-up measured by the Impact of Event Scale. Secondary outcomes are assessed for both breast cancer patients and partners. These outcomes are: general distress, symptoms of anxiety and depression, health-related quality of life and measures of dyadic adjustment, intimacy and partner involvement. Cancer-specific distress is also assessed for partners.

Eligible patients were women ≥ 18 years newly diagnosed with primary breast cancer, cohabiting with a male partner, having no previous cancer diagnoses, receiving no neo-adjuvant treatment, having no history of hospitalisation due to psychosis, and able to read and speak Danish. Partners were eligible if they could read and speak Danish and were ≥ 18 years.

**Discussion:**

This study investigates the effect of an attachment-oriented psychological intervention for breast cancer patients and their partners. The intervention has a theoretical framework and a strong design. If proven effective, this intervention would be helpful in optimising psychosocial care and rehabilitation of couples coping with breast cancer.

**Trial registration:**

ClinicalTrials.gov identifier: NCT01368380.

## Background

Breast cancer is a life-threatening disease, which can affect newly diagnosed women emotionally, socially and physically. For women in an intimate relationship, the partner is usually their main source of support throughout the trajectory of their cancer disease (Sjovall et al. 2009; Pistrang & Barker 1995). Thus it is important how the partner offers support as this may influence the patient’s level of distress and her adjustment to the disease (Hagedoorn et al. 2008; Waldrop et al. 2011). On the other hand, partners themselves may be affected in the same life domains as the patient (Sjovall et al. 2009; Pistrang & Barker 1995). Partners’ own needs will influence how they interpret the patient’s needs and how they support the patient. There is an increasing focus on couples’ adjustment to breast cancer, but there is a lack of couple-intervention studies with a clear theoretical basis and a strong design (Regan et al. 2012; Badr & Krebs 2013). This paper presents the development of the Hand in Hand couple-intervention (HiH) and the design of the randomised controlled trial (RCT) to test it.

We know that cancer patients and their intimate partners are at a significantly increased risk of developing symptoms of anxiety and depression. With regard to breast cancer, an observational cohort study with 222 breast cancer patients found that 48% of the women had at least one episode of depression or anxiety, or both, in the first year after diagnosis (Caroline et al. 2005). Further, a Danish cohort study found that breast cancer patients had a 14% prevalence of having depressive symptoms (Christensen et al. 2009). These differences might reflect time of assessment, assessment tool and the sample. Four different distress trajectories have been identified: a group of women not being distressed at any time-point (36.3%), women only being distressed during active treatment (33.3%), women being distressed only in the reentry and survivorship phase (15.2%), and women who are chronically distressed (15.2%) (Henselmans et al. 2010). Therefore, it is important to have continuous assessments and a representative sample. Further, a systematic review and meta-analysis found that both cancer survivors and their spouses had significantly higher prevalence of anxiety up to two years after diagnosis compared to healthy controls (Mitchell et al. 2013). The level of distress (including symptoms of anxiety and depression) may be affected by the cancer diagnosis, active treatment, and further by changes in roles, perceived support or lack thereof, and communication within the couple (Northouse et al. 1998; Fergus & Gray 2009).

Regarding partners of breast cancer patients, a cohort study following 20,538 partners of women with breast cancer concluded that the partners had a statistically significant hazard ratio of 1.39 of being hospitalised with an affective disorder up to 13 years after a partner’s cancer diagnosis compared to men with partners not being diagnosed with breast cancer (Nakaya et al. 2010). A longitudinal study of 92 couples facing breast cancer found no elevated self-reported distress in partners compared to a matched control group (Hinnen et al. 2008). The inconsistent results may reflect differences in measuring distress as self-reported or objective information, or how the populations were selected. Finally, a meta-analysis on distress in couples coping with cancer found a significantly modest positive correlation between patients’ and partners’ distress, substantiating the view that patients and partners mutually affect each other emotionally (Hagedoorn et al. 2008).

In addition to dealing with one’s own distress, members of couples confronted with cancer also need to deal with their partners’ distress (Hahn et al. 2005). Couples need to find a way to deal with each other’s emotions and the consequences for their relationship by offering and receiving support. Those who cope well with these challenges may find their relationships to be strengthened (Fergus & Gray 2009). Nonetheless, challenges that are not adequately coped with may increase levels of distress (Pielage et al. 2005) and these couples may benefit from psychological intervention aiming at increasing interdependent coping.

An increasing number of studies have examined different psychosocial interventions for cancer patients and their partners aimed at improving Quality of Life (QoL) and adjustment to the cancer diagnosis. Two systematic reviews with a total of 35 studies showed significantly small to moderate effect sizes regarding psychological, physical and relationship outcomes for both patients and partners (Regan et al. 2012; Badr & Krebs 2013). Nevertheless, the authors of both reviews pointed out that the results were influenced by conceptual and methodological limitations of the intervention studies, such as no specified theoretical framework, small sample sizes, high attrition rates and limited use of intention-to-treat analysis. The authors stated the need for well-designed studies that also investigate the integration of studies into clinical cancer care as well as cost-effectiveness. Furthermore, they stated that the content of an intervention should be flexible, hence making it possible to address couples’ present needs and to prepare patients and partners for psychological challenges they might experience later in the course of disease. A systematic review with 10 studies of psychological intervention for breast cancer patients and their partners (Brandao et al. 2014) concluded that these interventions appear to be effective, but these effects are influenced by similar limitations.

Based on the findings of previous research presented above, we developed a flexible intervention for couples facing breast cancer, addressing present psychosocial needs and helping to prepare for future challenges. Specifically, HiH is a psychological attachment-oriented couple intervention aimed to enhance dyadic adjustment through encouraging interdependent coping within the couples (e.g., discussing emotions and concerns and exchanging support). In turn, an interdependent coping style is expected to decrease symptoms of distress in initially distressed breast cancer patients and partners and to prevent distress in initially non-distressed breast cancer patients and partners. The remainder of this paper describes the theoretical framework and development of the HiH intervention and the design of the HiH RCT.

### Attachment theory

We use attachment theory as a theoretical framework to explain how couples respond and adjust to their new life situation after a breast cancer diagnosis (Burwell et al. 2006). The theory describes how attachment styles are developed in childhood as a result of the child’s repeated experiences of security in their caregiver interactions (Bowlby 1982). Attachment styles can be described as secure or insecure with regard to the view and valuation of one self and others.

Attachment theory explains how feeling secure and sharing feelings within intimate relationships help people to cope with threats and negative emotions. The presence of an available and responsive partner facilitates interdependent coping with threats such as breast cancer, whereas perceived or experienced unavailability of one’s partner disrupts coping and increases level of distress (Shaver et al. 2009). Attachment-oriented couples therapy aims to enable partners to perceive each other as a secure base and to encourage them to experience and share emotions (Bowlby 1978). Distressed couples may create new emotional experiences when they understand their partners’ attachment needs and underlying emotions. New emotional experiences occur when the couples interact in new ways that are based on new knowledge about each other (Johnson & Whiffen 1999; Adamson 2013).

### Primary aim

We developed an RCT to evaluate the effect of a couple intervention for breast cancer patients and their partners in the early treatment phase, comparing intervention in addition to usual care to usual care only. The aims of the intervention were to 1) reduce cancer-related and general distress, and symptoms of anxiety and depression in distressed and non-distressed cancer patients and partners, 2) increase health-related quality of life and post-traumatic growth of breast cancer patients and partners regardless of initial level of distress, and 3) increase dyadic adjustment in initially distressed and non-distressed breast cancer patients and partners.

## Methods

**Figure 1:**
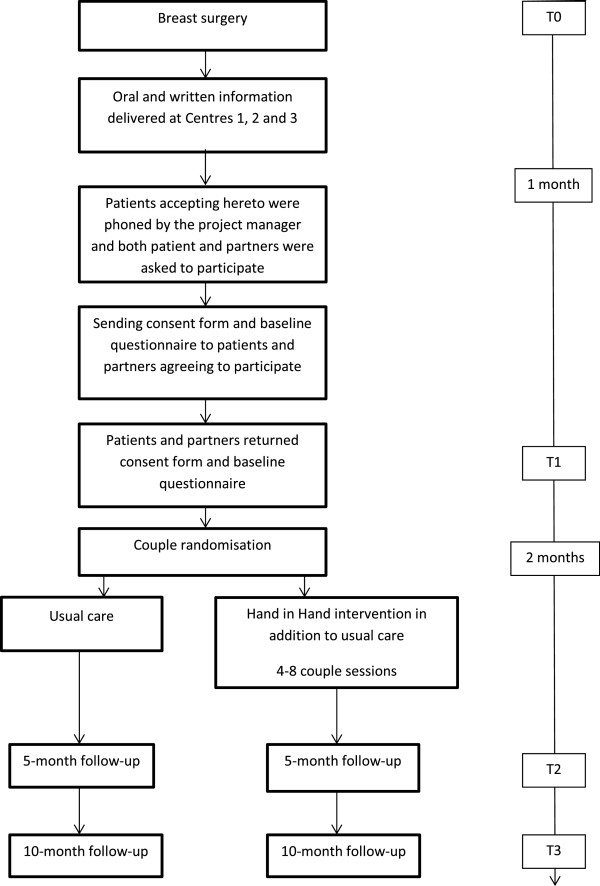
**Study design.**

This study is a multisite randomised controlled trial assessing the effectiveness of the Hand in Hand couple intervention (Figure 1). The HiH RCT will be analysed and reported in accordance with the CONSORT Statement (Boutron et al. 2008).

### Participants

#### Patients and partners

Eligible patients were women ≥ 18 years newly diagnosed with primary breast cancer, cohabiting with a male partner, having no previous cancer diagnoses, receiving no neo-adjuvant treatment, having no history of hospitalisation due to psychosis, and able to read and speak Danish. Partners were eligible if they could read and speak Danish and were ≥ 18 years. Patients and partners consulting any of the trial psychologists prior to inclusion could not participate.

### Recruitment

Participants were recruited at three Danish breast surgery departments: Ringsted Hospital (Centre 1), Odense University Hospital (Centre 2) from October 2011 to December 2012 and Herlev University Hospital (Centre 3) from April 2012 to January 2013. All centres treated patients from both rural and urban areas. Eligible patients received oral and written project information from a nurse or a healthcare worker during the medical discharge consultation after surgery or at the first following outpatient consultation. Patients were asked for permission to be phoned by the project manager within few weeks after the first outpatient consultation.

During phone contact, patients received a summary of the study and were given the opportunity to raise questions or concerns related to the study. Partners of patients who were interested in participation were then contacted and provided the same project information. Inclusion required written consent to participate and completed baseline questionnaire from both the patient and the partner.

### Randomisation

Randomisation was conducted following return of the signed informed consents and baseline questionnaires from patient and partner. Couples were randomised to the intervention or control group according to a computer-based randomisation procedure. The randomisation program was developed by a statistician of the research group and administered by an independent research assistant. The randomisation procedure was stratified on centres and each centre was block randomised in sequences calculated on the basis of each centre’s annual number of breast cancer surgeries. The block randomisation should ensure a more constant workload for the psychologists who had permanent positions in parallel with the project. All except the statistician were blinded to the block sizes and allocation sequence.

Couples were phoned by the project manager and informed about randomisation allocation. Obviously participants were not blinded with regard to the group assignment. Due to geographical reasons it was not possible to randomise the psychologists to centres.

### Usual care

Both the intervention and control group received usual care at the centres. Usual care involved oral and written information about frequent psychological reactions to receiving a cancer diagnosis. Two centres had additional offers. At *Centre 1* patients could be referred to psychological counselling with the in-house psychologist, i.e. a project psychologist. Emotionally distressed families with younger children could receive counselling by the in-house psychologist and a social worker from the Danish Cancer Society. *Centre 3* offered all breast cancer patients a free daytime seminar lasting three and a half day. Seminar participants received information about medical, psychological and social aspects of breast cancer.

### The Hand in Hand intervention

The intervention comprised 4-8 couple sessions with a psychologist in a period of approximately 3 months. The project group estimated that 4 couple sessions were sufficient to address emerging needs and dyadic distress in both initially distressed and non-distressed couples. The maximum of 8 couple sessions was chosen based on previous findings of an effect of Emotionally Focused Therapy (EFT) after 8 couple sessions for distressed couples (Denton et al. 2000; Johnson & Greenman 2006; Baucom et al. 1998). EFT helps couples create new emotional experiences and provide security to each other (Peluso & MacIntosh 2007). Consequently, 8 sessions should be sufficient for initially distressed couples to gain attachment security and new emotional experiences in the 3-month time frame (Denton et al. 2000). Participants could not receive more than 8 couple sessions. The first session lasted 90 minutes and the following sessions 60 minutes. Sessions were only conducted with attendance of both the patient and partner. The psychologists had no baseline information about the participants.

To avoid participants in the control group receiving counselling by trial psychologists outside the study, or participants in the intervention group receiving more than eight couple sessions, the trial psychologists could not be consulted outside the study until the 10-month follow-up. Regardless of allocation status, all participants were free to consult other psychologists during the time of study. At T3 all participants were asked if they have received any additional support and counselling (other than the intervention), and by whom this support has been provided.

An intervention manual was developed by the project manager and the trial psychologists. The manual comprised a general introduction to the background and the aim of the intervention, a short summary of attachment theory, and a description of issues to address in the couple sessions as well as during the first, the intermediate and the last couple sessions (Table 1). The psychologists decided how and when issues were addressed. It was stressed that the couple sessions should promote a safe and secure environment (Milberg et al. 2011; Garfield 2004) in which the couples could create new emotional experiences. In order to do so, the psychologist should address feelings of attachment insecurity, denial of emotional experience, unconscious suppression and rumination of threats (Shaver et al. 2009). The psychologists could organise home assignments, if they thought it to be beneficial for the couple.

**Table 1:** **Issues to address in hand in hand couple sessions**

Issues	Objective
Couples’ sense of attachment-related security	The couple is supported in focusing on their relationship strengths and attachment security, and supported in creating new emotional experiences
Level of individual emotional distress and needs	The couple is supported in verbalising their level of emotional distress and emotional needs, and feeling of attachment security
Knowledge of and experiences with cancer	The couple is supported in sharing their knowledge about breast cancer and their previous experiences with cancer, and how they influence their current situation
Psychological disorders	The couple is supported in verbalising present or previous psychological conditions, and if any, how they affect the couple in their current situation
Former stress-full life events	The couple is supported in verbalising previous experiences of emotional distress and their individual and dyadic adjustment in these distressed situations and how they can use these experiences in their current situation
Intimacy and sexual function	The couple is supported in verbalising needs and expectations related to intimacy and sexuality
Other stressors	The couple is supported in verbalising other factors that may affect the couple emotionally such as children’s and grandchildren’s reactions, work situation for them both, other diseases, economy and so forth

If couples randomised to the intervention group did not want to schedule the first session at their first contact with a psychologist, they could call back and schedule it within two months after randomisation.

#### First sessions

The primary task of the first session was to create a therapeutic alliance between the couple and the psychologist, and within the couple. Therapeutic alliance is a conscious, collaborative relationship between the therapist and clients (Garfield 2004). The psychologist should identify individual and dyadic distress and needs of the patient and the partner. The psychologist stressed that the focus of the sessions was on both patient and partner, and that the partner had an active and not only supportive role in the intervention.

#### Intermediate sessions

The content and number of intermediate couple sessions were flexible and individualised in accordance with couples’ needs. To apprehend the couples’ level of individual and dyadic distress, the psychologists addressed interactional patterns and emotional responses in the couples. For non-distressed couples focus should be on their relational strengths and how to prevent and manage distress in their current and future situation. As an addition to this focus, initially distressed couples should receive counseling in creating new emotional experiences.

#### Last sessions

Psychologists talked with the couples about what emotional reactions to expect in relation to the treatment phase, reentry phase and survivorship phase in relation to each couple’s experiences and level of distress. Further, it should be discussed how the couple could control and accept these reactions. Again couples discussed their experiences of attachment security and how they had integrated the received support and counseling into their daily lives.

### Psychologists

Four authorised psychologists were engaged, all of them experienced in health psychology, therapeutic counselling of cancer patients and couples, and familiar with attachment theory. The three psychologists affiliated to Centres 1 and 2 participated in the development of the intervention. All were instructed in adherence to the intervention manual, but received no additional training with regard to the intervention. To enhance protocol adherence, the psychologists completed a form after each session, indicating whether the focus had been on the individual patient or partner or on the couple, and what emotions and problems had been addressed.

### Ethics

Participants were informed that they at any given time and without reason could withdraw from the study. Hand in Hand was approved by the Health Research Ethics Committee System in Denmark; Record number S-20110100, By ClinicalTrials.gov; project number NCT01368380, and by the Danish Data Protection Agency; record number 2012-41-0392.

### Outcomes and data collection

Data were collected by questionnaires completed by patients and partners separately. Questionnaires and prepaid envelopes were mailed to patients and partners prior to randomisation (T1) and at the 5-month (T2) and 10-month (T3) follow-up.

#### Primary outcome

Primary outcome was change in patients’ cancer-related distress from T1 to T2, measured by Impact of Event Scale (IES) (Horowitz et al. 1979). IES is a validated scale with a total score and two subscales: “Intrusiveness” and “Avoidance”. IES is widely used for both breast cancer patients and partners (Manne et al. 2005a,^2008^; Scott et al. 2004).

#### Secondary outcomes

-  Symptoms of anxiety and depression assessed by the Hospital Anxiety and Depression Scale (HADS) (Zigmond & Snaith 1983)
-  General distress assessed by the Profile of Mood States – Short Form (POMS-SF) (DiLorenzo 1999)
-  Dyadic adjustment assessed by the Revised Dyadic Adjustment Scale (R-DAS) measuring consensus, satisfaction, and cohesion in the relationship (Busby et al. 1995)
-  Intimacy assessed with the Inclusion of Other in the Self Scale (IOS) (Aron et al. 1992)
-  Involvement of the partner assessed by a modified version of the Inclusion of Illness in the Self Scale (Aron et al. 1992)
-  Health-related quality of life assessed by Functional Assessment of Cancer Therapy – Breast (FACT–B) for patients (Brady et al. 1997; Northouse et al. 2012) and Functional Assessment of Chronic Illness Therapy–General (FACIT-G)(Brucker et al. 2005) for partners. For patient’s fatigue is assessed by the Functional Assessment of Cancer Therapy–Fatigue (FACT–F)(Yellen et al. 1997). To assess health economic effects, we measured health–related quality of life by the EuroQoL–5 dimensions (Sørensen et al. 2009).
-  Post–traumatic growth assessed by the Post–Traumatic Growth Inventory (PTGI) (Cordova & Andrykowski 2003).

#### Potential covariates

Clinical and demographic data included age, length of intimate relationship, education, stage of disease and treatment received for patients. These data were obtained from clinical databases, except length of intimate relationship, which was self-reported. The therapeutic alliance between participants in the intervention group and the psychologists was assessed by participants using the subscale “Bond” in the Working Alliance Inventory (WAI-SR) (Hatcher & Gillaspy 2006). Attachment style and dimensions of avoidance and anxiety were assessed by the Relationship Questionnaire (RQ)(Bartholomew & Horowitz 1991).

All outcomes were assessed at T1, T2 and T3; except post-traumatic growth, intimacy and involvement of the partner, which were not included at T1 and the “Bond” subscale of the WAI, which was only measured at T2. Couples, who had been in contact with the project manager but declined to participate, were asked to fill in a baseline questionnaire. The data will be used for a comparison of participants and non-participants.

### Sample size

Values of cancer-related distress on the Impact of Event Scale (IES–Total) range from 0 to 75. Based on prior intervention studies of breast cancer patients and their partners using IES–Total (Scott et al. 2004; Manne et al. 2005b), we estimated the mean of the patients to be 27 at baseline with a standard deviation (SD) of 16.5. Congruous to these previous studies, we considered a difference of 7 points clinically relevant. With a power of 0.90 and an alpha of 0.05, we aimed to include 220 couples.

### Statistical methods

The primary outcome being change in breast cancer patients’ cancer-related distress between T1 and T2 will be analysed with a linear regression, adjusted for baseline. Secondary outcomes will be analysed by means of multilevel analysis. Secondary analysis will be performed for selected variables and the effect over time on distress will be analysed. Factors that can affect level of distress will be investigated and adjusted for. Data on patients and partners as individuals will be analysed with multilevel techniques. Modified ITT analysis will be performed with a clear description of exactly who was included in each analysis.

## Discussion

To our knowledge HiH is the first psychological attachment-oriented couple intervention, targeting both initially distressed and non-distressed breast cancer patients and partners, that has been tested in a randomised controlled trial setting. Moreover, the focus on interdependent coping in the early treatment phase is a unique aspect. Conversely to other psychological interventions for patients and partners facing breast cancer, the HiH intervention used a semi-structured protocol, allowing adjustment of the intervention with regard to the number and content of the sessions.

We developed an intervention for couples who are distressed in the early breast cancer trajectory and non-distressed couples who may become distressed during the breast cancer trajectory. The aim was to reduce distress. The number of 4 to 8 sessions was estimated to be adequate to help couples to gain attachment security and to create new emotional experiences, thereby reducing and preventing distress.

Furthermore, we know that usual care varies across centres. However, we took great care to ask all participants at T3 if they had received any additional support and counselling (other than the intervention), and by whom this support has been provided. This information will be helpful in the interpretation of the results.

We have included therapeutic alliance and attachment-related anxiety and avoidance as potential covariates. Thereby we can ascertain potential moderating effects in the relationship between the participants and the psychologists. The measure of attachment-related anxiety and avoidance can help us to understand, if for example patients high on attachment-related anxiety benefit more from the intervention compared to patients low on attachment-related anxiety.

Results from previous intervention studies of breast cancer patients and their partners have been substantially influenced by methodological limitations such as small sample sizes, large attrition rates, inadequate description of attrition rates, and lack of specific randomisation procedures (Regan et al. 2012; Badr & Krebs 2013). We took the challenge to design and conduct a multi-centre, randomised, controlled trial that overcomes these limitations.

Furthermore, the HiH intervention addressed both distressed and non-distressed breast cancer patients and partners. We planned an intervention addressing attachment security and promoting new emotional experiences in a safe environment. A limitation of our design is that we compared the HiH intervention in addition to usual care with usual care only. It may be difficult to interpret the results with regard to the effect of the intervention, because a possible effect may be due to the mere fact that the intervention makes it possible for the couple to benefit from having leisure time together in a stressful situation.

To enhance protocol adherence, we made a treatment fidelity checklist to measure if the session had been performed in compliance with the intervention guide.

Some patients and/or partners declined to participate, because they felt overwhelmed by their new life situation and ongoing treatment or did not have the time to participate. Therefore, our sample might not be representative of primary breast cancer patients and their partners in general. We will address this by comparing characteristics of participants and non-participants.

By December 2013, the HiH study had succeeded in including 199 couples. Our sample size of 220 couples was calculated with a 0.90 power. Due to the fact that the inclusion at Centre 3 was delayed, we redid the sample size calculation with a 0.80 power. Based on this calculation, we wanted to include 166 couples. To take into account a risk of attrition of 20% we included 199 couples.

To conclude, receiving attachment-oriented psychological counselling in the early treatment phase is expected to reduce distress and to improve dyadic adjustment and health-related quality of life in breast cancer patients and their partners. If proven effective, this intervention would be helpful in optimising psychosocial care and rehabilitation of couples coping with breast cancer.

## Abbreviations

HiH: Hand in hand
RCT: Randomised controlled trial
QoL: Quality of life
CONSORT: Consolidated standards of reporting trials
EFT: Emotionally focused therapy
IES: Impact of event scale
HADS: Hospital anxiety and depression scale
POMS-SF: Profile of moods scale – short form
R-DAS: Revised dyadic adjustment scale
IOS: Inclusion of other in the self scale
FACT-B: Functional assessment of cancer therapy – breast
FACIT-G: Functional assessment of chronic illness therapy–general
FACT-F: The functional assessment of cancer therapy–fatigue
PTGI: Post traumatic growth inventory
WAI-SR: Working alliance inventory-short revised
RQ: Relationship questionnaire.

## References

[CR1] Adamson NA (2013). Emotionally focused therapy with couples facing breast cancer: a theoretical foundation and descriptive case study. Journal of Psychosocial Oncology.

[CR2] Aron A, Aron EN, Smollan D (1992). Inclusion of other in the self scale and the structure of interpersonal closeness. Journal of Personality and Social Psychology.

[CR3] Badr H, Krebs P (2013). A systematic review and meta-analysis of psychosocial interventions for couples coping with cancer. Psycho-Oncology.

[CR4] Bartholomew K, Horowitz LM (1991). Attachment styles among young adults: a test of a four-category model. Journal of Personality and Social Psychology.

[CR5] Baucom DH, Shoham V, Mueser KT, Daiuto AD, Stickle TR (1998). Empirically supported couple and family interventions for marital distress and adult mental health problems. Journal of Consulting and Clinical Psychology.

[CR6] Boutron I, Moher D, Altman DG, Schulz KF, Ravaud P (2008). Extending the CONSORT statement to randomized trials of nonpharmacologic treatment: Explanation and elaboration. Annals of Internal Medicine.

[CR7] Bowlby J (1978). Attachment theory and its therapeutic implications. Adolescent Psychiatry.

[CR8] Bowlby J (1982). Attachment and loss: retrospect and prospect. American Journal of Orthopsychiatry.

[CR9] Brady MJ, Cella DF, Mo F, Bonomi AE, Tulsky DS, Lloyd SR, Deasy S, Cobleigh M, Shimoto G (1997). Reliability and validity of the functional assessment of cancer therapy-breast quality-of-life instrument. Journal of Clinical Oncology.

[CR10] Brandao T, Schulz MS, Matos PM (2014). Psychological intervention with couples coping with breast cancer: a systematic review. Psychology and Health.

[CR11] Brucker PS, Yost K, Cashy J, Webster K, Cella D (2005). General population and cancer patient norms for the functional assessment of cancer therapy-general (FACT-G). Evaluation & the Health Professions.

[CR12] Burgess C, Cornelius V, Love S, Graham J, Richards M, Ramirez A (2005). Depression and anxiety in women with early breast cancer: five year observational cohort study. BMJ.

[CR13] Burwell SR, Brucker PS, Shields CG (2006). Attachment behaviors and proximity-seeking in cancer patients and their partners. Journal of Couple & Relationship Therapy.

[CR14] Busby DM, Christensen C, Crane DR, Larson JH (1995). A revision of the dyadic adjustment scale for use with distressed and nondistressed couples: construct hierachy and multidimensional scales. Journal of Marital and Family Therapy.

[CR15] Christensen S, Zachariae R, Jensen AB, Vaeth M, Moller S, Ravnsbaek J, von der Maase H (2009). Prevalence and risk of depressive symptoms 3-4 months post-surgery in a nationwide cohort study of Danish women treated for early stage breast-cancer. Breast Cancer Research and Treatment.

[CR16] Cordova MJ, Andrykowski MA (2003). Responses to cancer diagnosis and treatment: posttraumatic stress and posttraumatic growth. Seminars in Clinical Neuropsychiatry.

[CR17] Denton WH, Burleson BR, Clark TE, Rodriguez CP, Hobbs BV (2000). A randomized trial of emotion-focused therapy for couples in a trainin clinic. Journal of Marital and Family Therapy.

[CR18] Fergus KD, Gray RE (2009). Relationship vulnerabilities during breast cancer: patient and partner perspectives. Psychooncology.

[CR19] Garfield R (2004). The therapeutic alliance in couples therapy: clinical considerations. Family Process.

[CR20] Hagedoorn M, Sanderman R, Bolks HN, Tuinstra J, Coyne JC (2008). Distress in couples coping with cancer: a meta-analysis and critical review of role and gender effects. Psychological Bulletin.

[CR21] Hahn EA, Holzner B, Kemmler G, Sperner-Unterweger B, Hudgens SA, Cella D (2005). Cross-cultural evaluation of health status using item response theory. Evaluation & the Health Professions.

[CR22] Hatcher RL, Gillaspy JA (2006). Development and validation of a revised short version of the working alliance inventory. Psychotherapy Research.

[CR23] Henselmans I, Helgeson VS, Seltman H, de Vries J, Sanderman R, Ranchor AV (2010). Identification and prediction of distress trajectories in the first year after a breast cancer diagnosis. Health Psychology.

[CR24] Hinnen C, Ranchor AV, Sanderman R, Snijders TAB, Hagedoorn M, Coyne JC (2008). Course of distress in breast cancer patients, their partners, and matched control couples. Annals of Behavioral Medicine.

[CR25] Horowitz M, Wilner N, Alvarez W (1979). Impact of event scale: a measure of subjective stress. Psychosomatic Medicine.

[CR26] Johnson SM, Greenman PS (2006). The path to a secure bond: emotionally focused couple therapy. Journal of Clinical Psychology.

[CR27] Johnson SM, Whiffen VE (1999). Made to measure: adapting emotionally focused couple therapy to partners’ attachment styles. Clinical Psychology: Science and Practice.

[CR28] Manne SL, Ostroff JS, Winkel G, Fox K, Grana G, Miller E, Ross S, Frazier T (2005). Couple-focused group intervention for women with early stage breast cancer. Journal of Consulting and Clinical Psychology.

[CR29] Manne S, Winkel G, Ostroff J, Grana G (2005). Partner unsupportive responses, avoidant coping, and distress among women with early stage breast cancer: patient and partner persepctives. Health Psychology.

[CR30] Manne SL, Winkel G, Rubin S, Edelson M, Rosenblum N, Bergman C, Hernandez E, Carlson J, Rocereto T (2008). Mediators of a coping and communication-enhancing intervention and a supportive counseling intervention among women diagnosed with gynecological cancers. Journal of Consulting and Clinical Psychology.

[CR31] Milberg A, Wåhlberg R, Jakobsson M, Olsson EC, Olsson M, Friedrichsen M (2011). What is a ‘secure base’ when death is approaching? A study applying attachment theory to adult patients’ and family members’ experiences of palliative home care. Psycho-Oncology.

[CR32] Mitchell AJ, Ferguson DW, Gill J, Paul J, Symonds P (2013). Depression and anxiety in long-term cancer survivors compared with spouses and healthy controls: a systematic review and meta-analysis. Lancet Oncology.

[CR33] Nakaya N, Saito-Nakaya K, Bidstrup PE, Dalton SO, Frederiksen K, Steding-Jessen M, Uchitomi Y, Johansen C (2010). Increased risk of severe depression in male partners of women with breast cancer. Cancer.

[CR34] Northouse LL, Mood DW, Schafenacker A, Kalemkerian G, Zalupski M, LoRusso P, Hayes DF, Hussain M, Ruckdeschel J, Fendrick AM, Trask P, Ronis DL, Kershaw T (2012). Randomized clinical trial of a brief and extensive dyadic intervention for advanced cancer patients and their family caregivers. Psycho-Oncology.

[CR35] Northouse LL, Templin T, Mood D, Oberst M (1998). Couples adjustment to breast cancer and benign breast disease: a longitudinal analysis. Psycho-Oncology.

[CR36] Peluso PR, MacIntosh H (2007). Emotionally focused couples therapy and individual psychology: a dialogue across theories. Journal of Individual Psychology.

[CR37] Pielage SB, Luteijn F, Arrindell WA (2005). Adult attachment, intimacy and psychological distress in a clinical and community sample. Clinical Psychology & Psychotherapy.

[CR38] Pistrang N, Barker C (1995). The partner relationship in psychological response to breast cancer. Social Science & Medicine.

[CR39] Regan T, Lambert S, Girgis A, Kelly B, Kayser K, Turner J (2012). Do couple-based interventions make a difference for couples affected by cancer?: A systematic review. BMC Cancer.

[CR40] Scott JL, Halford KW, Ward BG (2004). United we stand? The effects of a couple-coping intervention on adjustment to early stage breast or gyneological cancer. Journal of Consulting and Clinical Psychology.

[CR41] Shaver PR, Mikulincer M, Lavy S, Cassidy J, Vangelisti AL (2009). Understanding and Altering Hurt Feelings: An Attachment-Theoretical Perspective on the Generation and Regulation of Emotions. Feeling Hurt in Close Relationships.

[CR42] Sjovall K, Attner B, Lithman T, Noreen D, Gunnars B, Thome B, Olsson H (2009). Influence on the health of the partner affected by tumor disease in the wife or husband based on a population-based register study of cancer in Sweden. Journal of Clinical Oncology.

[CR43] Sørensen J, Davidsen M, Gudex C, Pedersen KM, Brønnum-Hansen H (2009). Danish EQ-5D population norms. Scandinavian Journal of Public Health.

[CR44] Waldrop DP, O’Connor TL, Trabold N (2011). Waiting for the other shoe to drop: distress and coping during and after treatment for breast cancer. Journal of Psychosocial Oncology.

[CR45] Yellen SB, Cella DF, Webster K, Blendowski C, Kaplan E (1997). Measuring fatigue and other anemia-related symptoms with the Functional Assessment of Cancer Therapy (FACT) measurement system. Journal of Pain and Symptom Management.

[CR46] Zigmond AS, Snaith RP (1983). The hospital anxiety and depression scale. Acta Psychiatrica Scandinavia.

[CR47] The pre-publication history for this paper can be accessed here:http://www.biomedcentral.com/2050-7283/2/19/prepub

